# A charged tail on anti-α-Synuclein antibodies does not enhance their affinity to α-Synuclein fibrils

**DOI:** 10.1371/journal.pone.0308521

**Published:** 2024-08-29

**Authors:** Inga Petersen, Ana Godec, Farahnaz Ranjbarian, Anders Hofer, Claudio Mirabello, Greta Hultqvist

**Affiliations:** 1 Department of Pharmacy, Uppsala University, Uppsala, Sweden; 2 Department of Medical Biochemistry and Biophysics, Umeå University, Umeå, Sweden; 3 Department of Physics, Chemistry and Biology, National Bioinformatics Infrastructure Sweden, Science for Life Laboratory, Linköping University, Linköping, Sweden; Louisiana State University Health Sciences Center, UNITED STATES OF AMERICA

## Abstract

The aggregation of α-Synuclein (αSyn) is strongly linked to neuronal death in Parkinson’s disease and other synucleinopathies. The spreading of aggregated αSyn between neurons is at least partly dependent on electrostatic interactions between positively charged stretches on αSyn fibrils and the negatively charged heparan sulphate proteoglycans on the cell surface. To date there is still no therapeutic option available that could halt the progression of Parkinson’s disease and one of the major limitations is likely the relatively low proportion of αSyn aggregates accessible to drugs in the extracellular space. Here, we investigated whether a negatively charged peptide tail fused to the αSyn aggregate-specific antibodies SynO2 and 9E4 could enhance the antibodies’ avidity to αSyn aggregates in order to improve their potential therapeutic effect through inhibiting cell-to-cell spreading and enhancing the clearance of extracellular aggregates. We performed ELISAs to test the avidity to αSyn aggregates of both monovalent and bivalent antibody formats with and without the peptide tail. Our results show that the addition of the negatively charged peptide tail decreased the binding strength of both antibodies to αSyn aggregates at physiological salt conditions, which can likely be explained by intermolecular repulsions between the tail and the negatively charged C-terminus of αSyn. Additionally, the tail might interact with the paratopes of the SynO2 antibody abolishing its binding to αSyn aggregates. Conclusively, our peptide tail did not fulfil the required characteristics to improve the antibodies’ binding to αSyn aggregates. Fine-tuning the design of the peptide tail to avoid its interaction with the antibodies’ CDR and to better mimic relevant characteristics of heparan sulphates for αSyn aggregate binding may help overcome the limitations observed in this study.

## Introduction

The aggregation of α-synuclein (αSyn) in synaptic terminals is the common characteristic of synucleinopathies—a group of neurodegenerative disorders including Parkinson’s disease (PD), dementia with Lewy bodies, and multiple system atrophy [1]. In its healthy form, αSyn is monomeric in an equilibrium between a cytosolic, intrinsically disordered state and an α-helical membrane-bound state [2–5]. αSyn is predominantly located in the presynaptic terminals where it is considered to be involved in regulating vesicle trafficking and neurotransmitter release [6]. Though the role of αSyn aggregation in synucleinopathies, as to whether it is a symptom or a cause of disease, is still debated [6], there is a strong body of evidence showing neurotoxic effects of αSyn aggregates [7–11]. Numerous factors have been found to induce αSyn aggregation in vitro [2, 12–14], all of which are thought to create conditions which can promote a transient structure of αSyn that is self-association-prone and thus results in the formation of initial small αSyn oligomers [15]. These oligomers can grow bigger, convert into protofibrils and fibrils or remain non-fibrillar ‘off-pathway’ aggregates [16, 17].

αSyn oligomers are commonly considered soluble and non-fibrillar but have been reported with a broad range of molecular masses, diverse morphologies and different degrees of ß-sheet content [11, 16, 18–21]. In contrast, αSyn fibrils are well defined by their cross-β-sheet structure and their unbranched, elongated straight or twisted shape [22, 23].

αSyn oligomers are referred to as the most toxic species of αSyn aggregates and have been shown to exert their toxicity to cells in multiple ways, many of which involve their binding to or insertion into membranes [8, 24]. Toxicity has also been observed with αSyn fibrils, where the toxic effects are mostly ascribed to oligomeric species released by the fibrils’ ends [8]. Additionally, αSyn fibrils play a major role in seeding αSyn aggregation by releasing oligomeric species from fibril ends as well as catalyzing the conversion of αSyn monomers into misfolded species on the fibrils’ surface [8, 25]. Furthermore, αSyn fibrils are considered the primary species responsible for spreading αSyn pathology via cell-to-cell transfer [26–28]. Despite the predominant intracellular location of αSyn, both monomeric and aggregated species are also found to be released from neurons into the extracellular space by exocytosis [29, 30] or directly transmitted to neighbouring cells via exosomes or through tunnelling nanotubes [28, 31, 32]. Extracellular αSyn can continue to aggregate and seed aggregation in the extracellular space [33] and extracellular αSyn aggregates can be taken up into neighbouring neurons [34, 35]. The uptake of αSyn fibrils has been observed to be dependent on endocytosis by interactions with membrane-associated heparan sulfates (HS), e.g. heparan sulfate proteoglycans (HSPGs) [36–38] or with specific receptors [39–41]. HS also appear to modulate fibrillation, co-fibrillate, and stabilize fibrils not only in the case of αSyn [38] but also of many other amyloid proteins [42–46]. The interaction of HS with amyloid fibrils is of electrostatic nature [47] and has been suggested to depend on the overall negative charge of HS rather than a specific sulfation pattern [38]. With the highly repetitive, parallel packing of αSyn polypeptides in the amyloid fibril core, it is easy to imagine that identical charges align and form long stretches of positive or negative charge, where the positively charged stretches may represent large potential HS binding sites [38].

Different studies have shown promising effects using HS mimetics as therapeutics to reduce amyloid-β (Aβ) cytotoxicity and promote clearance in both *in vitro* and *in vivo* Alzheimer’s disease models [48, 49]. However, as HS have a large number of different ligands involved in numerous physiological functions, HS mimetic therapeutics possibly have a high risk for off-target binding. In contrast to Aβ, which is present at relatively high amounts in the extracellular space, targeting αSyn aggregation is particularly challenging due to its predominantly intracellular location. Hence, to therapeutically targeting αSyn pathology, limited to the extracellular αSyn pool, it is even more important to have a particularly high avidity and specificity towards αSyn aggregates in order to avoid sequestration of the drug by αSyn monomers or other off-target binding.

Here we aimed to test whether a negatively charged peptide ‘tail’ fused to αSyn-specific antibodies could enhance the antibodies’ avidity to αSyn fibrils by binding tightly to αSyn fibrils in a similar fashion as HS, but with the high specificity of the antibodies’ paratopes. We tested this strategy with the two αSyn aggregate-specific antibodies 9E4 and SynO2, both as full, bivalent antibody and as a monovalent format. Our results indicate that the negatively charged tail improved the binding of the bivalent 9E4 format to αSyn fibrils only at low-salt conditions (no added NaCl), whereas the tail improved the binding of the monovalent 9E4 format to αSyn fibrils only when the temperature was increased to 37°C (instead of room temperature). In the case of SynO2, the negatively charged tail reduced the antibodies binding strength in all tested conditions.

## Results

### Protein design and characterization of size and thermal stability

**Fig 1:**
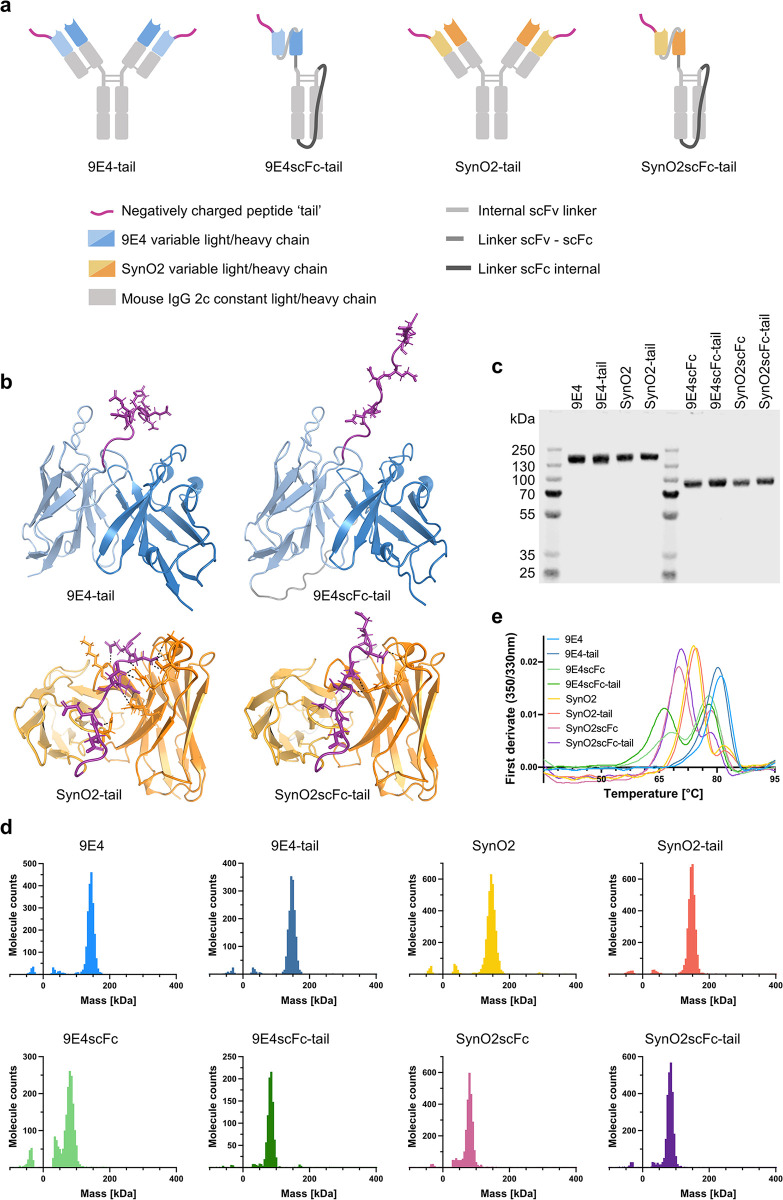
Antibody design and characterization of size and thermal stability. (a) Schematic representation of the bivalent and monovalent formats of the antibodies 9E4 and SynO2 with a negatively charged tail fused to the light chain N-terminus. (b) Alphafold2 structure predictions of the negatively charged tail (purple) fused to the light chain N-terminus of 9E4 (blue) and SynO2 (orange). Only the variable antibody domains are shown as part of the full antibody (left side) or with a linker connecting the light and heavy chains forming an scFv (right side) as part of the monovalent antibody format. Light chains are colored in light, heavy chains are colored in dark blue/orange, respectively. Black dashed lines indicate predicted polar interactions between the charged tail and SynO2 and SynO2scFc. Images were created in PyMOL [51]. (c) SDS-PAGE with Coomassie staining of purified antibodies using 1 μg protein/lane under non-reducing conditions. The complete gel can be seen in S1 Fig. (d) Mass distribution of purified antibodies measured at protein concentrations of 9–14 nM by mass photometry, confirming the purity and expected molecular mass of each antibody. Small peaks that appeared symmetrically at both -40 kDa and 40 kDa are most likely buffer impurities. Raw data can be found in S1 Table. (e) Thermal stability measurements of purified antibodies by their intrinsic fluorescence at 350 nm and 330 nm over a temperature ramp from 35°C to 95°C. Measured at protein concentration of 0.13 mg/ml. Peaks in the first derivate of the 350 nm/330 nm ratio represent major unfolding events. For raw data see S2 Fig and S2 Table.

To mimic the charge properties of HS with a peptide, a sequence consisting of aspartic acid and glutamic acid residues spaced by glycines was chosen and recombinantly fused with a three amino acid linker to the N-terminus of the antibodies’ light chain (Fig 1A). A monovalent antibody format was designed by fusing a single chain fragment variable (scFv) to a single chain fragment constant (scFc) IgG2c [50], where the peptide was fused to the N-terminus of the scFv of 9E4 or SynO2 (Fig 1A). The structures of the variable chains of both bivalent and monovalent antibodies with the peptide tail were predicted using AlphaFold2 (Fig 1B). The obtained structures suggested a potential interaction of the negatively charged tail with several amino acids in the complementary-determining regions (CDRs) of SynO2, whereas the tail was free when fused to 9E4 (Fig 1B). To test the charge-dependence of this predicted interaction, structures of the antibodies’ scFvs fused to a positively charged tail was predicted which did not show any interaction with the variable regions of either 9E4 or SynO2 (S7 Fig). All antibodies were produced in Expi293 cells and purified with high purity (Fig 1C and S1 Fig) from the cell medium by protein G purification yielding 8–18 mg protein per L cell culture for the full antibodies and 0.4–7 mg protein per L cell culture for the scFc fusions. Mass photometry measurements confirmed the correct size and homogeneity of all antibodies (Fig 1D and S1 Table). The antibodies’ thermal stability was evaluated by the change of intrinsic fluorescence at 350 nm and 330 nm over a temperature ramp from 35°C to 95°C. The unfolding profiles with peaks representing major unfolding events differ between 9E4 and SynO2 as well as between the full antibody and the monovalent format, but the addition of the negatively charged peptide did not appear to affect the thermal stability of any of the antibodies (Fig 1E, S2 Fig and S2 Table).

### Determining differences in binding strength of the antibodies by ELISA

**Fig 2:**
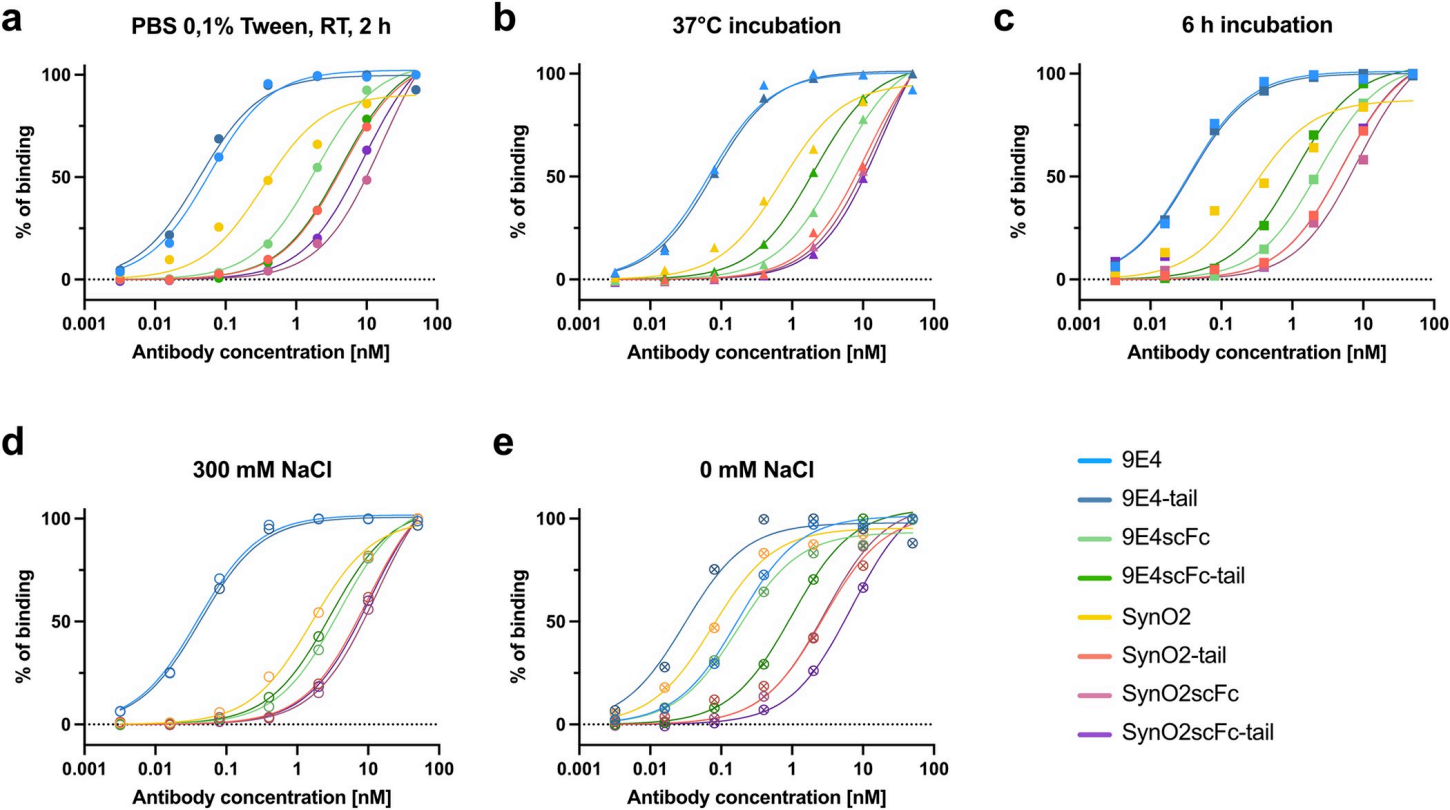
Indirect ELISA to test the binding of 9E4, 9E4scFc, SynO2, and SynO2scFc, all with and without a negatively charged peptide ‘tail’, to αSyn fibril coating. The antibodies’ binding strength was tested in different conditions such as (a) “standard conditions” PBS 0.1% Tween, at RT for 2 h, (b) incubation at 37°C, (c) incubation for 6 h, (d) incubation in phosphate buffer 0.1% Tween with 300 mM NaCl, (e) incubation in phosphate buffer 0.1% Tween with 0 mM NaCl. ELISA curves were normalized to maximum binding signal of each antibody. Non-linear regression curves were created using a “one site–specific binding” model in GraphPad Prism. For non-normalized data see S3 and S4 Figs. Raw data can be found in S3 Table.

To test whether the negatively charged tail could enhance the antibodies’ binding strength towards αSyn fibrils, we performed indirect enzyme-linked immune assays (ELISA) with a coating of sonicated preformed αSyn fibrils (PFF), on which we incubated the antibodies in different conditions. As expected due to avidity with a bivalent compared to a monovalent antibody format, the full antibodies showed much higher binding strengths than the monovalent scFc formats under all conditions (Fig 2, S3 and S4 Figs and S3 Table).

For both the full SynO2 antibody and the SynO2scFc, the addition of the tail appeared to cause a strong decrease in binding strength under all tested conditions (Fig 2). In contrast, the full 9E4 antibody and 9E4scFc respectively with and without tail behaved rather similar under standard conditions (incubation in phosphate-buffered saline (PBS) 0.1% tween for 2 h at room temperature (RT)) (Fig 2A). Considering that the interaction between the charged peptide tail and the target might establish with different kinetics than with the antibodies’ CDRs, we performed ELISAs with incubating the antibodies on the target at increased temperature (37°C) or increased time (6 h). Indeed, we observed a slightly stronger binding of 9E4scFc-tail compared to 9E4scFc without tail under both conditions, whereas there was no difference in binding strength between the full antibody 9E4 with or without tail (Fig 2B, 2C).

Considering that the negatively charged tail may potentially interact with charged residues in the antibodies’ CDRs, as proposed for the SynO2 antibodies by the predicted protein structure (Fig 1B), we further tested different salt concentrations in the ELISA incubation buffer. Here, salt ions could screen charged residues on both the antibody and the tail and thus block potential interactions. With 300 mM NaCl as high salt condition, the binding strength of all antibodies decreased (Fig 2D). With no NaCl added to the incubation buffer, however, the binding strength of all antibodies except for 9E4 increased (Fig 2E). Hence, 9E4-tail achieved the highest apparent affinity in the 0 mM NaCl condition.

Both SynO2 and 9E4 are reported to be specific for αSyn aggregates, which may include both oligomers and fibrils. By applying a relatively long sonication to the αSyn fibrils, we likely achieved a strong fragmentation which may also result in the release of oligomeric species to some degree. To investigate whether the degree of fibril fragmentation affects the antibodies’ binding strength, we sonicated the αSyn fibrils for only 2 min instead of 10 min, resulting in less fibril fragmentation and thus larger fibrils. Using those longer fibrils as target in our standard ELISA set-up resulted in an overall decrease in binding signal of all antibodies (S4 Fig).

As HS-binding is a common property among many amyloid proteins, we also tested whether the negatively charged tail increased the antibodies’ binding to Aβ fibrils as one example of ‘off-target’ amyloid. However, in our ELISA standard condition we did not detect any increase in binding with any of the tail-antibodies (S5 Fig and S4 Table).

## Discussion

The relative low abundance of αSyn aggregates in the extracellular space is only one of the major limitations in targeting the propagation of αSyn pathology as a therapeutic strategy in synucleinopathies. Thus, very high affinity and specificity of αSyn targeting drugs are particularly important to achieve the highest effect possible despite the low target concentration.

One way to enhance an antibody’s binding strength to a multimeric target like αSyn aggregates is by increasing the antibody’s valency, as we have previously shown with multivalent formats of SynO2 and of the Aβ protofibril-specific antibody mAb158 [52–54]. In the present study, we followed a similar strategy of increasing the antibodies’ valency to αSyn aggregates, but here by fusing short, negatively charged peptides to the antibodies, which were intended to mimic the electrostatic interaction between αSyn and HS. As HS have been suggested to be involved in multiple mechanisms of amyloid pathogenesis such as fibrillation and cell-to-cell propagation [36], we hypothesized that a drug mimicking this interaction could interfere with those mechanism by competing with HS for the binding of αSyn aggregates and thus inhibit HS-mediated cell-to-cell spreading and potentially enhance clearing of extracellular αSyn aggregates.

The therapeutic potential of HS or heparin mimetics have indeed been investigated as in the context of inflammation [55, 56], viral infections [57], cancer [58, 59], as well as amyloidosis [48, 49]. HS mimetics have been shown to bind soluble Aβ and reduce the plaque load in an Alzheimer’s disease mouse model [48, 49]. Heparin itself on the other hand has been shown to induce amyloid fibrillation, however, without causing pathology but instead reducing toxic effects in cell models [60, 61] possibly by sequestering the more toxic oligomeric species [62].

Off-target binding may be a problem with HS mimetics against amyloids due to the rather unspecific charge-dependent interaction type between HS and amyloids [38, 47]. We therefore suggest here a combined antibody-HS-mimetic approach, where the antibody provides a high specificity towards αSyn aggregates while the negatively charged peptide tail adds valency to increase the avidity. However, we could not observe any prominent tail-mediated avidity effect when testing the antibodies in ELISA against αSyn fibrils. Instead, we observed a strong decrease in binding strength of SynO2-tail and SynO2scFc-tail to αSyn fibrils compared to SynO2 alone. There may be different reasons that could explain those results. First of all, the design of our peptide tail focused only on creating a high density of negatively charged amino acids to mimic ionic properties of HS, which may be a too primitive attempt of mimicking HS properties. It was previously concluded that the interaction of amyloids with HS is primarily dependent on electrostatic interactions and not on particular sulfation patterns [38]. However, HS are highly heterogenous and the exact factors that cause the binding of αSyn fibrils to HS are not fully elucidated. Thus, it is possible that negative charges alone are not sufficient for amyloid binding but that some degree of sulfation is still required or at least beneficial for stronger binding to amyloid fibrils. Peptides with site-specific sulfation [63, 64] could be one option to study the role of sulfation in binding αSyn aggregates further. Further, the distance and position of negative charges are also likely to play a role in amyloid fibril binding. A distance of ~5 Å between the parallelly aligned αSyn polypeptide chains in αSyn fibrils (S6A Fig) suggests that a similar distance between negative charges on HS or HS mimetics may be favorable for interacting with the positively charged residues in αSyn fibrils [65]. The distance between the charged residues in our peptide tail appears to be in the range of 7–9 Å (S6B Fig) which may be slightly too large to match the charge distribution on αSyn fibrils.

αSyn also contains many negatively charged amino acids, some of which also align in the β-sheets of the fibril core. Additionally, the C-terminus of αSyn is highly negatively charged and thus could potentially repel the binding of our negatively charged peptide, especially considering that both 9E4 and SynO2 supposedly recognize the C-terminus of αSyn [66, 67]. Our observation that the binding of SynO2 to αSyn fibrils seems to be much more negatively affected by the tail than the binding of 9E4 may be due to different epitopes of SynO2 and 9E4 possibly causing stronger or weaker electrostatic repulsion by nearby negative charges in αSyn.

Lastly, using Alphafold2 structure predictions of our antibodies with the negatively charged tail we found that the tail potentially interacts with amino acids in the CDRs of SynO2 whereas there was no such interaction found in the case of 9E4. Thus, the peptide tail blocking the CDRs in SynO2 may explain the drastic loss in binding strength observed with ELISA. Those predicted structures may not be 100% accurate considering the flexible nature of both CDRs and the peptide tail [68] but we believe that those structure predictions may indicate tendencies in the proteins’ structure and can serve as a best guess. In an attempt to proof that electrostatic intramolecular interactions or intermolecular repulsion are in fact responsible for the results we see with ELISA, we tested different ionic strengths of the ELISA incubation buffer. With high salt (300 mM NaCl) or low salt (0 mM NaCl) conditions, we aimed to see a shift in binding strength of the antibodies with tail relative to the respective antibody without tail due to changes in the degree of ionic screening or a higher exposure of the tail [69]. As electrostatic interactions may also play a role in the CDRs’ binding to the epitope, screening those interactions by a high salt concentration may be the reason for the overall decrease in binding strength seen here with all antibodies. Accordingly, the opposite effect may happen at the low-salt concentration causing an overall increase in binding strength of all tested antibodies. However, at low-salt conditions, the apparent affinity of the 9E4-tail antibody outperformed 9E4, indicating that intermolecular repulsion between the tail and αSyn fibrils are likely responsible for the tail not having an effect on the antibody’s binding strength under physiological salt conditions. Hence, the negatively charged peptide tail can indeed act on enhancing the antibody’s binding to αSyn fibrils under low ionic strength conditions, which cause reduced intermolecular electrostatic repulsion [70]. Surprisingly, no effect in the low-salt condition was seen with the monovalent format 9E4scFc-tail, suggesting that a bivalent format is required for the tail to have an avidity enhancing effect.

In conclusion, we have shown here that the fusion of a negatively charged peptide to the N-terminus of SynO2 decreased the antibody’s apparent affinity to αSyn fibrils dramatically, which can likely be ascribed to intramolecular interactions of the tail with the antibody’s CDR or to repulsion between the tail and clustered negative charges on αSyn aggregates. The antibody 9E4 was less negatively affected by the fusion with the tail, however 9E4-tail did not achieve the goal of enhancing the antibody’s avidity to αSyn fibrils under physiological salt concentrations, likely due to electrostatic repulsion between the tail and negative charges on αSyn fibrils. Optimizing the peptide tail design towards more HS-mimicking properties including net charge, charge distribution and sulfation may help with improving its effect on enhancing and antibodies affinity and specificity to αSyn fibrils under physiological conditions.

## Methods

### Protein design and production

The variable light (VL) and heavy (VH) chain sequences of SynO2 (patent number EP2961774B1) and 9E4 (patent number US8609820B2) were recombinantly fused to IgG2c constant light and heavy chains to produce a full antibody. A monovalent antibody format was created with scFv of SynO2 and 9E4 in VL-VH orientation with an internal (G_4_S)_3_ linker. The scFv was fused via a linker with the sequence APGSGGGSAPG to the N-terminus of an IgG2c scFc [50]. The negatively charged peptide sequence GEDGEDGEDPGS was fused with a PGS linker to the N-terminus of the antibodies’ light chain or scFv. All antibody sequences were cloned into pcDNA3.4 vectors (GeneArt, Regensburg, Germany) and transfected into Expi293 cells as described previously [71]. Antibodies were purified from the cell culture supernatant using a protein G column (Cytiva GE17-0404-01) using a gradient elution with 0–100% of 0.7% acetic acid. The eluted antibodies were concentrated on a 30K MWCO Amicon Ultra-15 centrifugal filter unit (Millipore UFC9030) and dialyzed to PBS in a SnakeSkin™ dialysis tubing (Thermo Scientific 88243) or using a 7K MWCO Zeba spin desalting column (Thermo Scientific 89892). The protein purity and size was validated by SDS-PAGE with Coomassie staining and mass photometry.

### Protein structure prediction

Protein structures were predicted using AlphaFold2 [72] with either the monomer_ptm model preset or the multimer model v2.3 preset [73] for the prediction of the monovalent or bivalent antibody, respectively. For the monovalent antibody, only the structure of the scFv was predicted. For the bivalent antibody, only the structure of the variable domains was predicted. Five predictions were generated with the monomer model and 25 predictions with the multimer model. Default AlphaFold databases were used. The structures presented in this paper are the highest ranking model after Amber relaxation. Potential polar contacts between the peptide tail and the antibody were identified using the find—polar contacts command in PyMOL [51]. All images showing protein structures were created in PyMOL [51].

### SDS-PAGE

The purified antibodies were analysed by SDS-PAGE to assess their purity. Protein concentrations were determined by the protein’s absorbance at 280 nm measured on a DS-11 spectrophotometer (DeNovix, Wilmington, USA) together with the protein’s molecular mass and extinction coefficient, which were calculated from the protein sequence using the ProtParam tool on the ExPASy server [74]. 1 μg of each purified antibody was mixed with 1x LDS sample buffer (Invitrogen B0007) under non-reducing conditions and separated on a Bolt 4 to 12% Bis-Tris 1 mm protein gel (Invitrogen NW04125) at 80–100 V for 1–2 h. A pre-stained protein marker (Thermo Scientific 26619) was used as a molecular mass standard. Gels were stained in PAGE blue protein solution (Thermo Scientific 24620).

### Mass photometry

For further validation of the antibodies’ correct size and to detect antibody aggregation, mass photometry was carried out on a Refeyn 2MP instrument (Refeyn Ltd., Oxford, UK). After calibration with NativeMark Unstained Protein Standard (Thermo Scientific LC0725), antibodies were analyzed at a concentration of 9–14 nM. The method is based on the proportional relationship between the intensity of the light scattering signal generated by molecules touching the instrument’s glass surface and the molecule’s molecular mass [75].

### Tycho

The antibodies’ thermal stability was analysed using a Tycho nt.6 instrument (NanoTemper Technologies, Munich, Germany) at a concentration of 0.13 mg/ml. Here, a protein’s unfolding is measured through the change in its intrinsic fluorescence at 330 nm and 350 nm over a temperature ramp from 35°C to 95°C.

### Indirect ELISA

The antibodies’ affinities to αSyn fibrils or Aβ protofibrils were measured by indirect ELISA. αSyn fibrils at a concentration of 11.7 μM were sonicated for 10 min in a bath sonicator (Branson 5510) to generate a more homogeneous mixture of short fibrils. A high-binding 96-well plate (Sarstedt 82.1581.200) was coated with 50 μl/well 10 nM sonicated αSyn fibrils or 10 nM Aβ protofibrils in PBS overnight at 4°C. The next day, each plate was blocked with 1% bovine serum albumin (BSA) (Sigma-Aldrich A7030) in PBS for 2 h shaking at RT. Serial dilutions of each antibody were prepared in either standard ELISA incubation buffer (0.1% BSA and 0.05% Tween-20 in PBS), in 10 mM phosphate buffer pH 7.4 or in 10 mM phosphate buffer pH 7.4 with 300 mM NaCl. Each antibody dilution was added in duplicates to the plate and incubated either shaking for 2 h at RT, for 2 h at 37°C, or for 6 h at RT. A goat anti-mouse horseradish peroxidase (HRP)-conjugated antibody (Sigma-Aldrich 12–349) diluted 1:10,000 in standard ELISA incubation buffer was incubated on the plate for 1 h and antibody binding was detected by the colorimetric signal generated through the addition of K-blue aqueous TMB (Neogen 331177). The reaction was stopped by the addition of 1M sulphuric acid after 2 min and the absorbance at 450 nm was measured on a TECAN Spark plate reader (Tecan, Männedorf, Switzerland). The plate was washed with PBS with 0.05% Tween-20 between each incubation step of the protocol.

## Supporting information

S1 Fig Raw image of SDS-PAGE with Coomassie staining shown in Fig 1c. (TIF)

S2 Fig Raw data of intrinsic fluorescence intensity at 350 nm and 330 nm measured by Tycho. The fluorescence intensity ratio 350 nm/330 nm is plotted against the temperature.
(TIF)

S3 Fig Non-normalized binding curves of indirect ELISA shown in Fig 2. Indirect ELISA to test the binding of 9E4, 9E4scFc, SynO2 and SynO2scFc, all with and without a negatively charged peptide ‘tail’, to αSyn fibril coating. The antibodies’ binding strength was tested in different conditions such as (a) “standard conditions” PBS 0.1% Tween, at RT for 2 h, (b) incubation at 37°C, (c) incubation for 6 h, (d) incubation in phosphate buffer 0.1% Tween with 300 mM NaCl, (e) incubation in phosphate buffer 0.1% Tween with 0 mM NaCl. Raw data can be found in S3 Table.
(TIF)

S4 Fig Non-normalized binding curves of indirect ELISA shown in Fig 2 and S3 Fig sorted by antibody. Indirect ELISA to test the binding of 9E4, 9E4scFc, SynO2 and SynO2scFc, all with and without a negatively charged peptide ‘tail’, to αSyn fibril coating. The antibodies’ binding strength was tested in different conditions such as “standard conditions” PBS 0.1% Tween at RT for 2 h (filled circle), incubation at 37°C (filled triangle), incubation for 6 h (filled square), incubation in phosphate buffer 0.1% Tween with 300 mM NaCl (empty circle), incubation in phosphate buffer 0.1% Tween with 0 mM NaCl (empty circle with cross), coating with longer PFF (sonicated for a shorter time) (inverted filled triangle). Raw data can be found in S3 Table.
(TIF)

S5 Fig Non-normalized binding curves of indirect ELISA with Aβ protofibril coating. Indirect ELISA to test the ‘off-target’ binding of 9E4, 9E4scFc, SynO2 and SynO2scFc, all with and without a negatively charged peptide ‘tail’, to Aβ protofibril coating under “standard conditions” (PBS 0.1% Tween at RT for 2 h). The Aβ protofibril-specific antibody mAb158 was used as a positive control. Raw data can be found in S4 Table.
(TIF)

S6 Fig Distances (in Å) of positive charges on αSyn fibrils and on the negatively charged tail. (a) Cross-β-sheet structure forming the core of αSyn fibrils (PDB ID 2N0A [76]) where charged residues align to long stretches of positive (red) or negative (blue) charge. The distance of 5 Å between positively charged residues of neighbouring αSyn chains is indicated with dashed lines. (b) Structure of the negatively charged peptide tail as predicted for the 9E4-tail antibody using Alphafold2. Glutamic acid and aspartic acid residues are shown as sticks. Distances measured between the negatively charged residues are indicated with dashed line. PyMOL [51] was used for distance measurements and to create the images in (a) and (b).
(TIF)

S7 Fig Alphafold2 structure predictions of a positively charged peptide tail (dark red) fused to the light chain N-terminus of 9E4 (blue) and SynO2 (orange). The sequence of the positively charged peptide is GKRGKRGKRPGS. Only the scFv domains are predicted with a linker connecting the light and heavy chains as part of the monovalent antibody format. Light chains are colored in light, heavy chains are colored in dark blue/orange, respectively. In contrast to the predicted structures of SynO2 with the negative tail (Fig 1b), no interactions between the variable antibody domains and the positively charged tail were predicted. Images were created in PyMOL [51].
(TIF)

S1 Table Raw data of mass distribution measurements by mass photometry shown in Fig 1d. (XLSX)

S2 Table Raw data of thermal stability analysis on the Tycho instrument shown in Fig 1e and S2 Fig. (XLSX)

S3 Table Raw data of indirect ELISAs shown in Fig 2, S3 and S4 Figs. (XLSX)

S4 Table Raw data of indirect ELISAs shown in S5 Fig. (XLSX)
