# Supplementary figures and images for: A charged tail on anti-α-Synuclein antibodies does not enhance their affinity to α-Synuclein fibrils
Source: PLoS One. 2024 Aug 29;19(8):e0308521. doi: 10.1371/journal.pone.0308521 (PMC11361660; doi:10.1371/journal.pone.0308521)

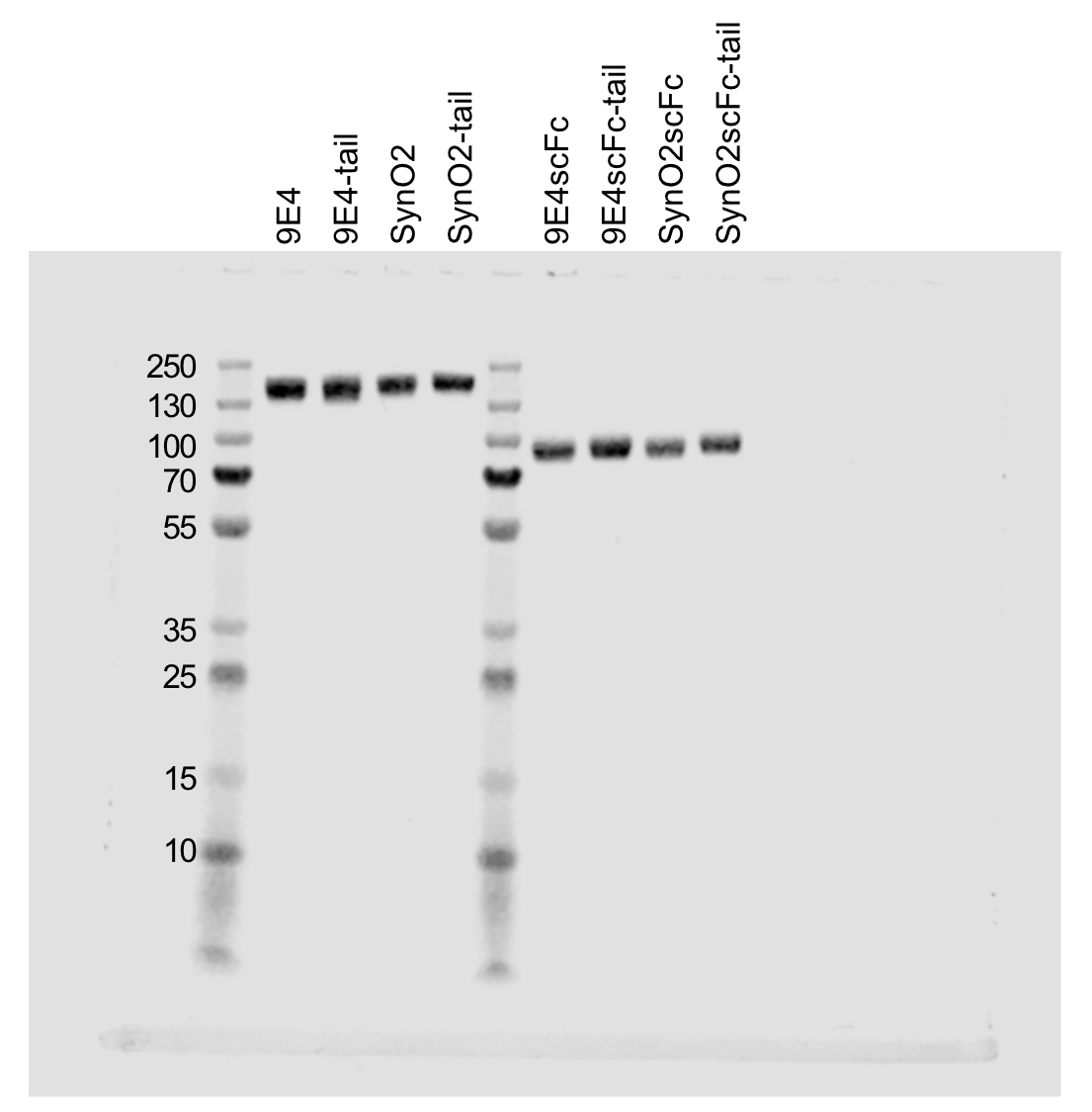

Supplement: S1 Fig — (TIF) [file pone.0308521.s001.tif]

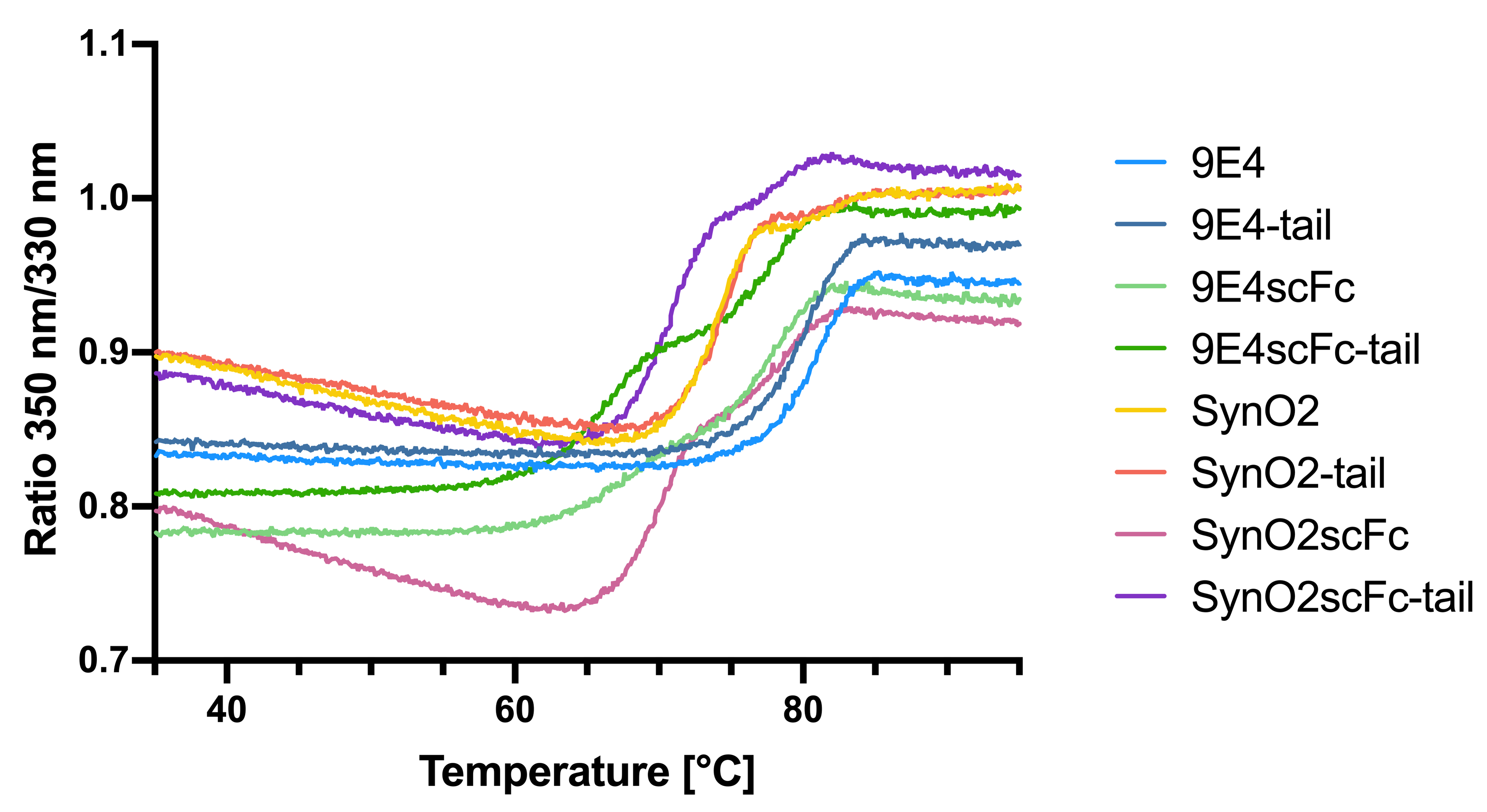

Supplement: S2 Fig — The fluorescence intensity ratio 350 nm/330 nm is plotted against the temperature. (TIF) [file pone.0308521.s002.tif]

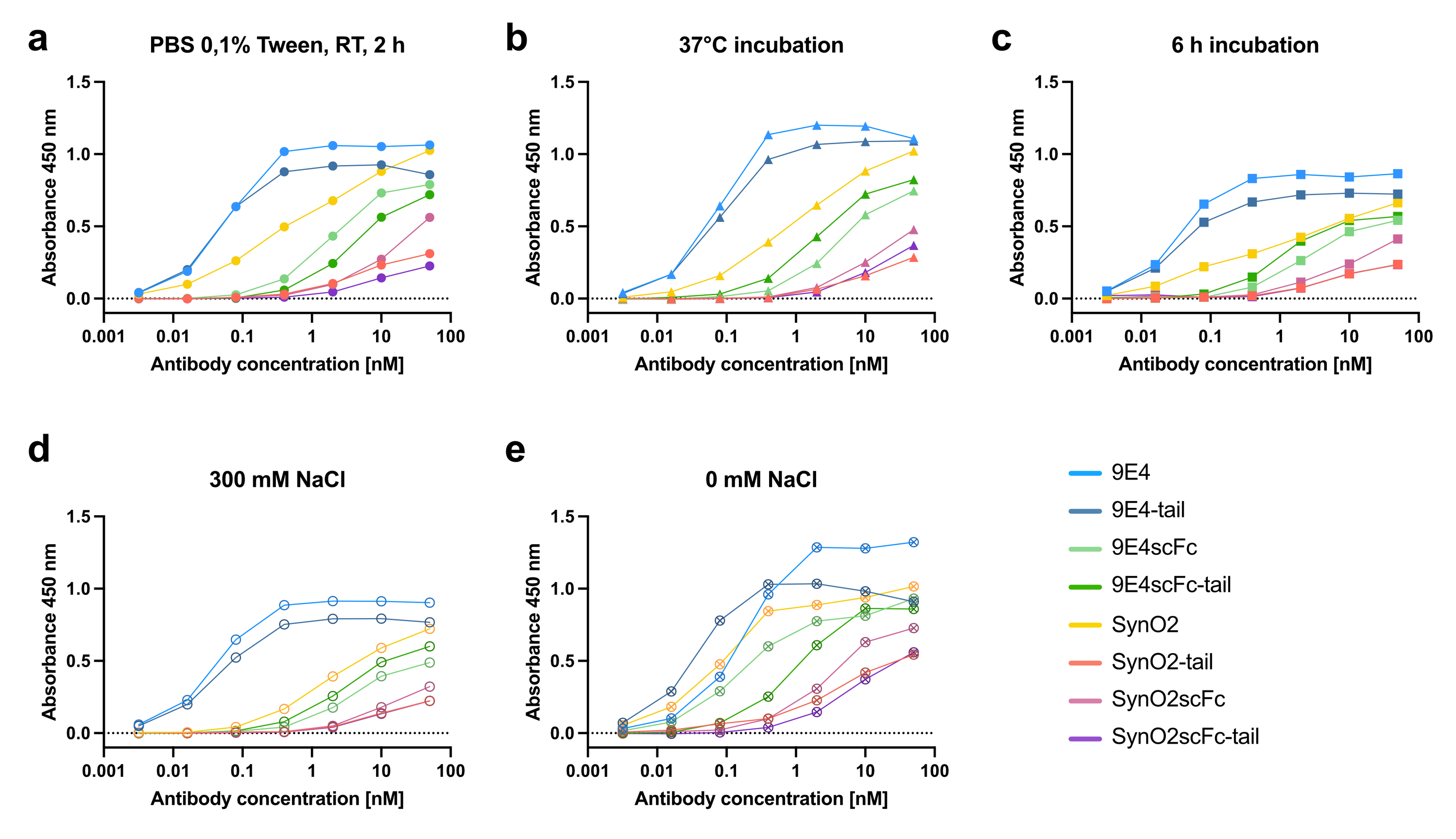

Supplement: S3 Fig — Indirect ELISA to test the binding of 9E4, 9E4scFc, SynO2 and SynO2scFc, all with and without a negatively charged peptide ‘tail’, to αSyn fibril coating. The antibodies’ binding strength was tested in different conditions such as (a) “standard conditions” PBS 0.1% Tween, at RT for 2 h, (b) incubation at 37°C, (c) incubation for 6 h, (d) incubation in phosphate buffer 0.1% Tween with 300 mM NaCl, (e) incubation in phosphate buffer 0.1% Tween with 0 mM NaCl. Raw data can be found in S3 Table. (TIF) [file pone.0308521.s003.tif]

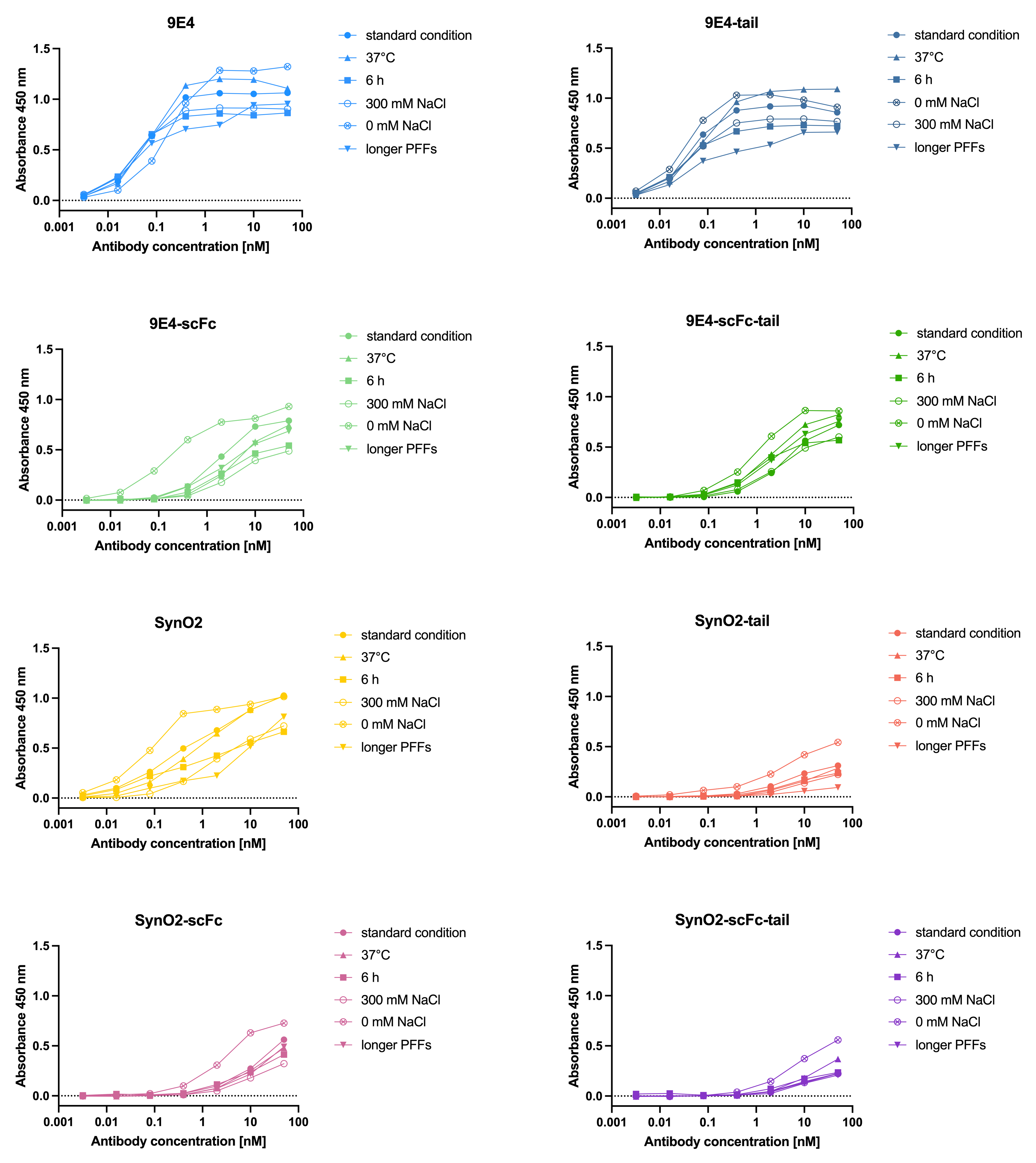

Supplement: S4 Fig — Indirect ELISA to test the binding of 9E4, 9E4scFc, SynO2 and SynO2scFc, all with and without a negatively charged peptide ‘tail’, to αSyn fibril coating. The antibodies’ binding strength was tested in different conditions such as “standard conditions” PBS 0.1% Tween at RT for 2 h (filled circle), incubation at 37°C (filled triangle), incubation for 6 h (filled square), incubation in phosphate buffer 0.1% Tween with 300 mM NaCl (empty circle), incubation in phosphate buffer 0.1% Tween with 0 mM NaCl (empty circle with cross), coating with longer PFF (sonicated for a shorter time) (inverted filled triangle). Raw data can be found in S3 Table. (TIF) [file pone.0308521.s004.tif]

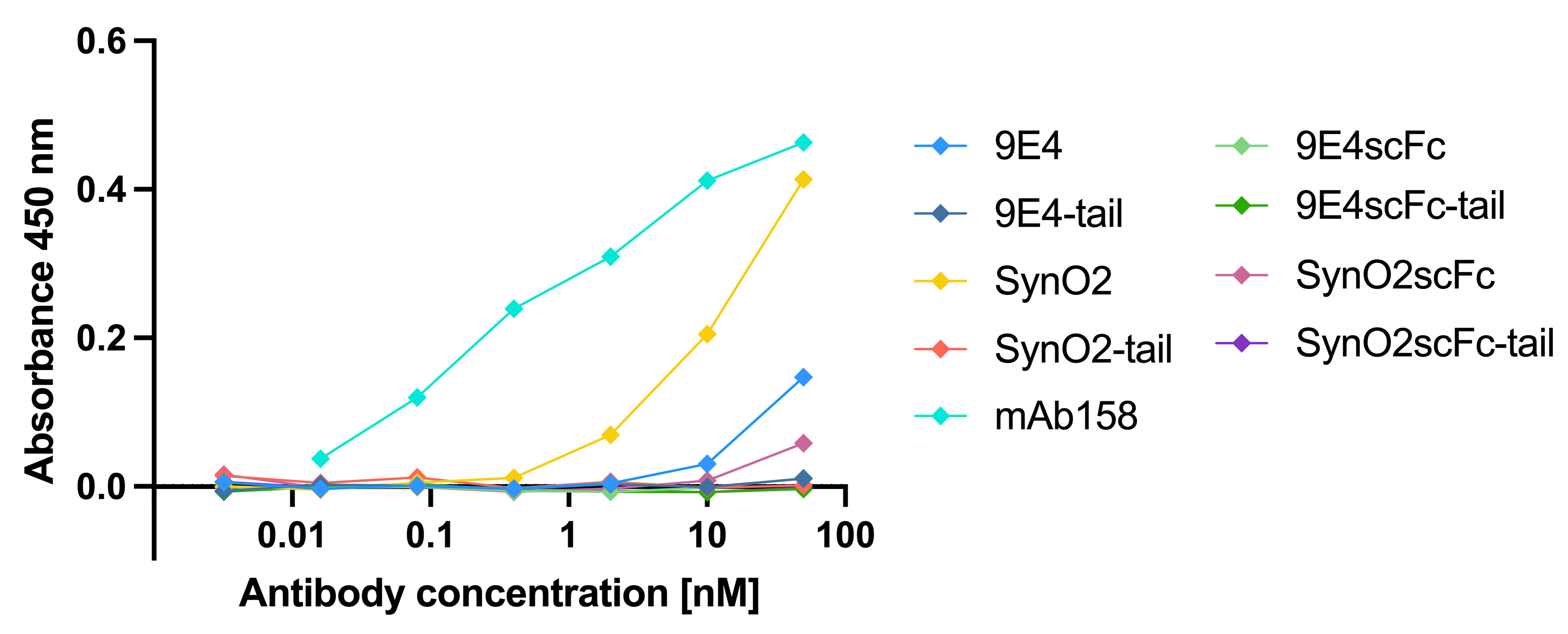

Supplement: S5 Fig — Indirect ELISA to test the ‘off-target’ binding of 9E4, 9E4scFc, SynO2 and SynO2scFc, all with and without a negatively charged peptide ‘tail’, to Aβ protofibril coating under “standard conditions” (PBS 0.1% Tween at RT for 2 h). The Aβ protofibril-specific antibody mAb158 was used as a positive control. Raw data can be found in S4 Table. (TIF) [file pone.0308521.s005.tif]

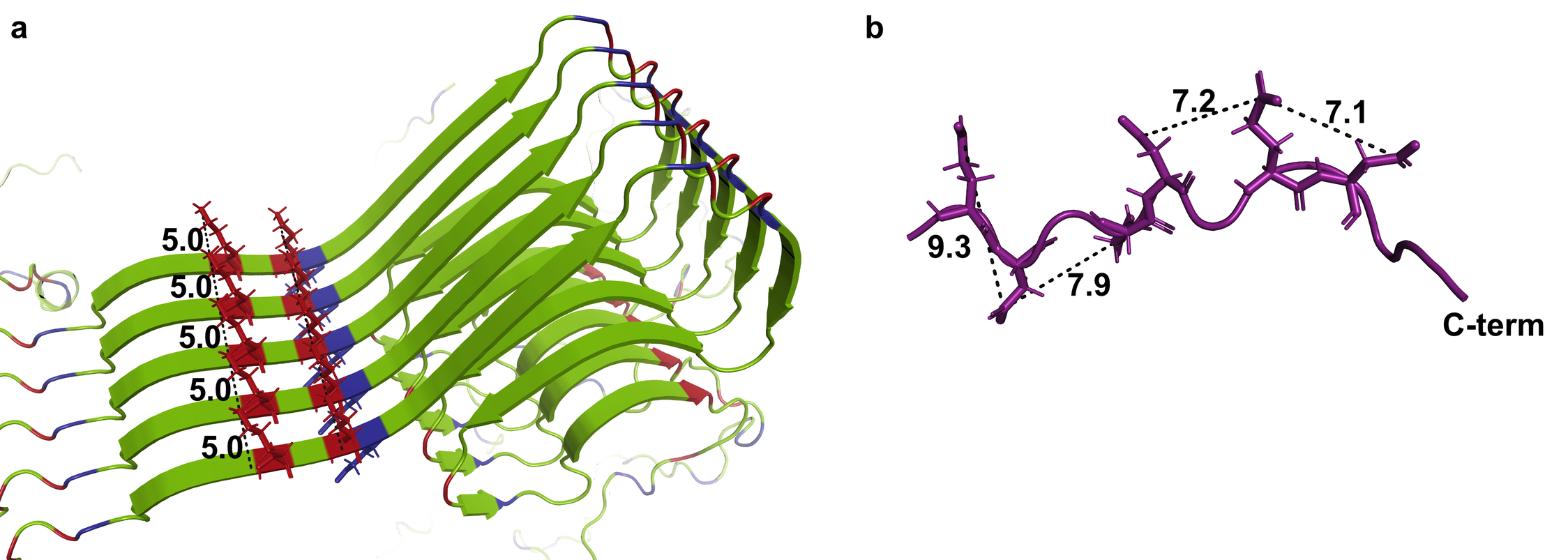

Supplement: S6 Fig — (a) Cross-β-sheet structure forming the core of αSyn fibrils (PDB ID 2N0A [76]) where charged residues align to long stretches of positive (red) or negative (blue) charge. The distance of 5 Å between positively charged residues of neighbouring αSyn chains is indicated with dashed lines. (b) Structure of the negatively charged peptide tail as predicted for the 9E4-tail antibody using Alphafold2. Glutamic acid and aspartic acid residues are shown as sticks. Distances measured between the negatively charged residues are indicated with dashed line. PyMOL [51] was used for distance measurements and to create the images in (a) and (b). (TIF) [file pone.0308521.s006.tif]

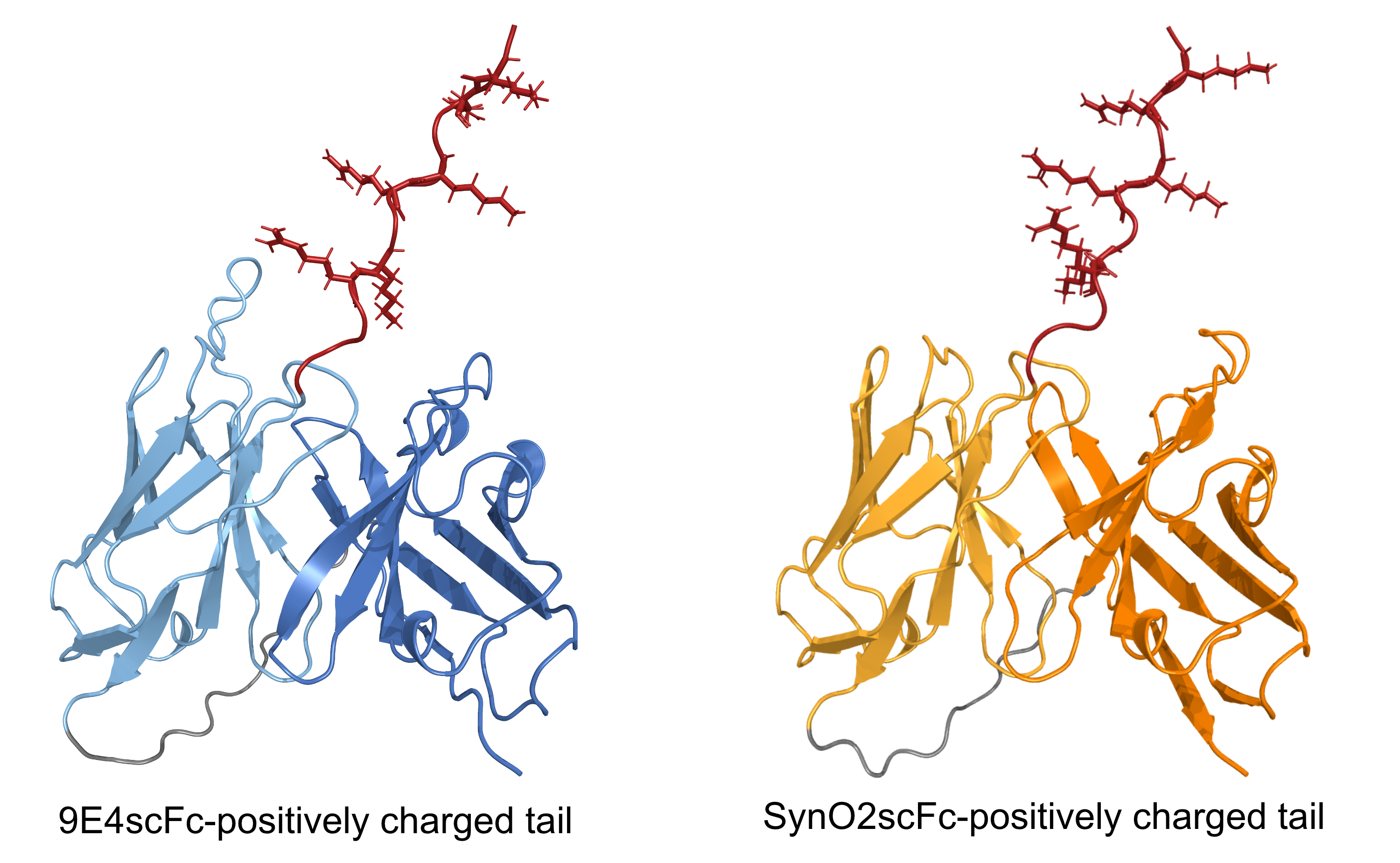

Supplement: S7 Fig — The sequence of the positively charged peptide is GKRGKRGKRPGS. Only the scFv domains are predicted with a linker connecting the light and heavy chains as part of the monovalent antibody format. Light chains are colored in light, heavy chains are colored in dark blue/orange, respectively. In contrast to the predicted structures of SynO2 with the negative tail (Fig 1b), no interactions between the variable antibody domains and the positively charged tail were predicted. Images were created in PyMOL [51]. (TIF) [file pone.0308521.s007.tif]
